# The ability of late pregnancy maternal tests to predict adverse pregnancy outcomes associated with placental dysfunction (specifically fetal growth restriction and pre-eclampsia): a protocol for a systematic review and meta-analysis of prognostic accuracy studies

**DOI:** 10.1186/s13643-020-01334-5

**Published:** 2020-04-08

**Authors:** Melanie Griffin, Alexander E. P. Heazell, Lucy C. Chappell, Jian Zhao, Deborah A. Lawlor

**Affiliations:** 1 NIHR BRC Reproductive and Perinatal Health Group, Oakfield House, Oakfield Grove, Bristol, BS8 2BN UK; 2 Tommy’s Maternal and Fetal Research Centre, Manchester Academic Health Science Centre, Division of Developmental Biology and Medicine, Faculty of Biology, Medicine and Health, The University of Manchester, 5th Floor (Research), St Mary’s Hospital, Oxford Road, Manchester, M13 9WL UK; 3 School of Life Course Sciences, King’s College London, 10th Floor, North Wing, St Thomas’ Hospital, Westminster Bridge Road, London, UK; 4 MRC Integrative Epidemiology Unit, University of Bristol, Bristol, BS8 2BN UK; 5 Population Health Science, Bristol Medical School, University of Bristol, Bristol, UK

**Keywords:** Pre-eclampsia, Small for gestational age (SGA), Fetal growth restriction (FGR), Neonatal growth restriction/growth restriction in the newborn (NGR), Stillbirth, Biomarker, Placenta, Ultrasound

## Abstract

**Background:**

Pre-eclampsia and being born small for gestational age are associated with significant maternal and neonatal morbidity and mortality. Placental dysfunction is a key pathological process underpinning these conditions; thus, markers of placental function have the potential to identify pregnancies ending in pre-eclampsia, fetal growth restriction, and the birth of a small for gestational age infant.

**Primary objective:**

To assess the predictive ability of late pregnancy (after 24 weeks’ gestation) tests in isolation or in combination for adverse pregnancy outcomes associated with placental dysfunction, including pre-eclampsia, fetal growth restriction, delivery of a SGA infant (more specifically neonatal growth restriction), and stillbirth.

**Methods:**

Studies assessing the ability of biochemical tests of placental function and/or ultrasound parameters in pregnant women beyond 24 weeks’ gestation to predict outcomes including pre-eclampsia, stillbirth, delivery of a SGA infant (including neonatal growth restriction), and/or fetal growth restriction will be identified by searching the following databases: EMBASE, MEDLINE, Cochrane CENTRAL, Web of Science, CINAHL, ISRCTN registry, UK Clinical Trials Gateway, and WHO International Clinical Trials Portal. Any study design in which the biomarker and ultrasound scan potential predictors have been assessed after 24 weeks’ gestation but before diagnosis of outcomes (pre-eclampsia, fetal growth restriction, SGA (including neonatal growth restriction), and stillbirth) will be eligible (this would include randomized control trials and nested prospective case-control and cohort studies), and there will be no restriction on the background risk of the population. All eligible studies will be assessed for risk of bias using the modified QUADAS-2 tool. Meta-analyses will be undertaken using the ROC models to estimate and compare test discrimination and reclassification indices to test calibration. Validation will be explored by comparing consistency across studies.

**Discussion:**

This review will assess whether current published data reporting either a single or combination of tests in late pregnancy can accurately predict adverse pregnancy outcome(s) associated with placental dysfunction. Accurate prediction could allow targeted management and possible intervention for high-risk pregnancies, ultimately avoiding adverse outcomes associated with placental disease.

**Systematic review registration:**

PROSPERO CRD42018107049

## Background

Pre-eclampsia and being born small for gestational age (SGA) are associated with significant maternal, fetal, and neonatal morbidity and mortality [1–3]. Despite the burden of disease associated with these conditions, in the UK, there has been little decline in their prevalence over the past two decades and pre-eclampsia remains a leading cause of maternal death worldwide [4]. Placental dysfunction plays a key role in the shared pathophysiology of these conditions [5–8]. Tests of placental function have been proposed as potential prognostic and diagnostic tools [9].

Delivery of a SGA infant is commonly defined as a birth weight under a specified threshold and includes both constitutionally small and growth-restricted infants, whereas growth restriction is more specifically defined as the failure of a fetus or infant to reach their full growth potential and can be diagnosed antenatally (fetal) or at birth (neonatal). The relationship between delivery of a SGA infant and adverse pregnancy outcome is likely secondary to the contribution of fetal (FGR) and/or neonatal growth restriction (NGR) within this group. Identifying growth restriction is complex, and study definitions vary for this outcome and are frequently misclassified. Delivery of a SGA infant is often seen as a surrogate for FGR and NGR and sometimes incorrectly used interchangeably with these definitions.

Most studies assessing maternal serum biomarkers of placental origin (e.g., pregnancy-associated plasma protein A (PAPP-A), alpha-fetoprotein (AFP)) to predict adverse pregnancy outcomes have tested in the first and/or second trimesters and have insufficient test accuracy for clinical use [10–12]. While combining biochemical with biophysical markers in the first and/or second trimesters appears to improve predictive ability, current evidence is insufficient for them to be recommended for use in clinical practice [8, 13–15].

Sampling later in pregnancy could provide greater predictive accuracy, as this is closer to disease onset (compared to first or second trimester sampling). This is supported by studies evaluating biomarkers associated with placental function in the third trimester [16–19]. There is most evidence to support the use of late pregnancy maternal tests for the prediction of pre-eclampsia, e.g., tests based upon placental growth factor (PlGF), but other studies also report benefits in high-risk and unselected populations for the prediction of delivering a SGA infant [18, 20]. Screening in late pregnancy using maternal tests to predict stillbirth is limited to a few small studies, and results are conflicting [20–22].

In an attempt to improve clinical outcomes, by targeted intervention for those at greatest risk, the most accurate late pregnancy prediction model for adverse pregnancy outcomes associated with placental dysfunction must be identified. Systematic reviews and meta-analyses assessing the predictive ability of late pregnancy tests (specifically maternal serum biomarkers) for conditions associated with placental dysfunction have not to date identified any accurate prediction tool whose use is beneficial in clinical practice [10, 23, 24]. However, several large studies reporting promising results for biomarkers in prediction of adverse pregnancy outcomes, particularly pre-eclampsia, have been published since the most recent of those previous systematic reviews [16, 17, 19, 20]. The aim of this review is to reevaluate the ability of late pregnancy tests to predict adverse pregnancy outcomes associated with placental dysfunction incorporating recent evidence.

A Cochrane diagnostic test accuracy review of biochemical tests of placental function versus ultrasound assessment of fetal size for stillbirth and SGA infants has already been undertaken (AH, lead author). Therefore, this review will primarily focus on prediction of pre-eclampsia and fetal and neonatal growth restriction (FGR and NGR) but will also include a search for any new studies, published subsequent to the period covered by the Cochrane review, predicting stillbirth and delivery of a SGA infant, so that a comprehensive up to date review of all evidence can be provided.

## Objective

The primary objective is to explore the predictive ability of late pregnancy (after 24 weeks’ gestation) maternal biochemical tests of placental function (including human placental lactogen (hPL), soluble fms-like tyrosine kinase-1 (sFlt-1), placental growth factor (PlGF), sFlt-1: PlGF ratio), and ultrasound parameters (including estimated fetal weight (EFW), umbilical and uterine artery Doppler pulsatility index, and amniotic fluid index) in isolation or in combination for adverse pregnancy outcomes associated with placental dysfunction, including pre-eclampsia, FGR, delivery of a SGA infant (more specifically NGR), and stillbirth.

## Methods/design

This protocol has been prospectively registered with the International Prospective Register of Systematic Reviews (PROSPERO; CRD42018107049) and will follow the preferred reporting items for systematic review and meta-analysis protocol (PRISMA-P) guidelines (Additional file 1). This includes details of inclusion and exclusion criteria, a data extraction tool, and analytical methods.

### Inclusion criteria

To be included, they must explore the use of biochemical tests of placental function and/or ultrasound parameters in pregnant women as accurate predictors of key outcomes. Specifically, studies must:

- Assess biochemical placental function tests and/or ultrasound parameters after 24 completed weeks’ gestation but before the occurrence of outcomes. The threshold of 24 weeks’ gestation for the assessment of the late pregnancy predictor (the focus of this review) has been selected to facilitate comparison with data soon to be reported in a Cochrane review, predicting stillbirth, and delivery of a SGA infant.
- Evaluate their ability to predict one or more of these outcomes: pre-eclampsia, stillbirth, FGR, delivery of a SGA infant (including NGR), diagnosed after the biomarker, or ultrasound scan measurements have been assessed.

Studies assessing the predictive accuracy of late pregnancy maternal tests have used varying definitions for SGA, FGR, and NGR. To avoid excluding any potential studies in this systematic review, studies using any definition of SGA, FGR, or NGR will be assessed. For analysis, some studies will be reclassified according to the outcome measures outlined in this protocol. If any study definitions differ from those we use in this protocol (see section on outcome measures), that study data will be analyzed separately to the main outcome measures.

Any study that fulfills these criteria will be included (this could for example, include randomized controlled trials, prospective cohort, or nested prospective case-control studies). There will be no restriction on the background risk of the population (i.e., unselected or high-risk antenatal populations), and studies of women of any age will be included. Placental function biomarkers in urine or blood can be measured using any assay technique and threshold for a positive result. Studies evaluating the predictive ability of any ultrasound measurement measured beyond 24 weeks’ gestation will be included; these could include uterine and/or umbilical artery Doppler pulsatility index, estimated fetal weight, and amniotic fluid index.

### Exclusion criteria

Studies including multifetal pregnancies and pregnancies complicated by major lethal fetal abnormality identified during pregnancy will be excluded, as these are independent risk factors for the pre-specified outcomes and it is reasonable to assume that concentrations of biomarkers would be different due to increased placental mass in multifetal pregnancies and altered trophoblast function in aneuploidy. Retrospective case-control and cross-sectional studies will be excluded.

### Outcome measures

The primary outcome measures are pre-eclampsia, fetal growth restriction (FGR), delivery of a small for gestational age (SGA) infant (including neonatal growth restriction (NGR)), and stillbirth, defined as:

I. Pre-eclampsia: New hypertension (> 140/90 mmHg) presenting after 20 weeks’ gestation and significant proteinuria (urinary protein:creatinine ratio > 30 mg/mmol or a validated 24-h urine collection > 300 mg protein or urine dipstick reading > 1+) or maternal organ dysfunction:
  - Renal insufficiency (creatinine > 90 μmol/L)
  - Liver involvement (elevated transaminases to twice normal concentration for pregnancy and/or severe right upper quadrant or epigastric pain)
  - Neurological complications (examples include eclampsia, altered mental status, blindness, stroke, or more commonly hyperreflexia when accompanied by clonus, severe headaches when accompanied by hyperreflexia, persistent visual scotomata)
  - Hematological complications (thrombocytopenia platelets < 100,000/μL, DIC, hemolysis) [25] [note that we will only include pre-eclampsia diagnosed after measurement of the predictor—so beyond 24 weeks’ gestation]
  Any studies using other definitions of pre-eclampsia will be considered but analyzed and reported separately.
II. FGR: Abdominal circumference (AC) or estimated fetal weight (EFW) < 3rd gestation specific centile or AC/EFW < 10th gestation specific centile combined with uterine artery Doppler pulsatility index (UtAPI) > 95th centile and/or umbilical artery Doppler pulsatility index (UAPI) > 95th centile [26]
III. SGA: A birth weight classified as small for gestational age (SGA) within the study. Study definitions will be documented if this differs to birth weight ≤ 10th gestation specific centile, using population or customized birth weight centiles
IV. NGR: A birth weight ≤ 3rd gestation specific centile, on population or customized growth charts or at least 3 out of 5 of the following:

- Birth weight < 10th centile
- Head circumference < 10th centile
- Length < 10th centile
- Prenatal diagnosis of FGR
- Maternal pregnancy information (e.g., hypertension or pre-eclampsia) [27]

V. Stillbirth: An infant born with no signs of life (that was not a planned termination of pregnancy)

Prior to analysis, studies reporting different labels for the same definitions as our outcome measures (such as severe SGA for neonatal growth restriction and intrauterine growth restriction for fetal growth restriction) will be reclassified according to the definitions specified above.

Any studies that include outcomes assessed before 24 weeks’ gestation will be initially included (i.e., at the full paper screen stage), and we will attempt to use information in the paper and/or from contact with authors to obtain estimates restricted to outcomes after 24 weeks’ gestation.

### Search strategy

A detailed search strategy with no language or date restrictions will search the following sources: EMBASE, MEDLINE, Cochrane CENTRAL, Web of Science, CINAHL, ISRCTN registry, UK Clinical Trials Gateway, WHO International Clinical Trials Portal, and specialist abstract and conference proceeding resources (Web of Science Conference Proceedings Citation Index and British Library’s ZETOC). A draft for the search strategy to be used is detailed in Additional file 2.

In addition, information on ongoing studies, unpublished research and research reported in the grey literature will be sought. We will report our search terms as an appendix to published studies.

### Study reviews and appraisals

MG and JZ will independently review the titles and abstracts of all studies identified by the search strategy to identify potentially relevant studies. Full text versions of all potentially relevant studies will be sought and independently assessed for inclusion by MG and JZ following the pre-specified inclusion criteria stated in the methods. If there is disagreement, the opinion of a third review author (DAL) will be sought. Reasons for study exclusion will be documented.

### Data extraction

Data will be extracted independently by MG and JZ and collected using a customized data collection form. Recorded data will include participant characteristics, presence of one or more of our pre-specified outcome measures (including definitions used), type of biomarker assay or test combinations (including thresholds for a positive test), and processing and storage of samples before use. Data related to pregnancy outcome but additional to our pre-specified outcome measures will also be collected, including mode of delivery, maternal complications, and admission to neonatal intensive care unit.

Sensitivity and specificity of a prognostic test will be extracted when available; otherwise, true-positive (TP), false-positive (FP), true-negative (TN), and false-negative (FN) counts will be collected. For studies reporting data using multiple thresholds for a test, a 2 × 2 table of the above counts will be extracted at each threshold. If samples are reported at multiple time points within a study, then each will be considered individually. If these data are not available, we will extract odds ratios and/or area under (AUC) receiver operating characteristic (ROC) curves. If study data, critical to our proposed analysis, is unclear or absent, the relevant authors will be contacted for clarification.

The risk of bias in each study will be evaluated using the modified QUADAS-2 tool [28]. Two review authors (MG and JZ) will independently assess each study and assign either “yes,” “no,” or “unclear” to each of the criteria stated in the modified QUADAS-2 tool. This information will be summarized graphically in the final review document. Studies will not be excluded from the main meta-analysis on the basis of risk of bias.

### Statistical analysis

For those studies where a 2 × 2 table can be extracted, test specific sensitivity will be calculated as TP/(TP + FN) and specificity will be calculated as TN/(TN + FP). Sensitivity will be plotted against specificity to construct separate receiver operating characteristic (ROC) curves for each primary outcome to determine test discrimination and assess for heterogeneity. A hierarchical summary ROC (HSROC) model will be used to pool the results to estimate summary sensitivity and specificity with associated confidence intervals and a ROC curve [29–31]. The HSROC model takes account of both the within- and between-study variability by inclusion of a study specific random effect that allows for heterogeneity between studies. If a study reports multiple test thresholds, the data related to the most frequently reported threshold across all studies will be included in the meta-analysis. We will explore the use of more advanced methodology, which allows pooling of multiple points per study [32, 33]. We will calculate net reclassification in each study as a measure of calibration and pool these using appropriate methods for proportions [34]. When test specific sensitivity and specificity data or a 2 × 2 table data are not available, we will perform meta-analysis of odds ratios and/or AUC [35].

### Exploration of heterogeneity

We will quantify the amount of heterogeneity using the between studies standard deviation in logit sensitivity and logit specificity. If sufficient data are available, we will explore potential sources of heterogeneity using subgroup analyses/meta-regression. Key study characteristics that we intend to explore include risk of bias, study design, and population characteristics.

Once we have completed the systematic review and initial summary of completed studies, we will update this protocol in relation to these subgroup analyses. All statistical analysis will be carried out in the statistical package Stata version 14 (College Station, TX, USA).

## Discussion

This review will provide essential data necessary to evaluate the ability of late pregnancy tests in isolation and combination to predict adverse pregnancy outcomes related to placental dysfunction. It will be the first comprehensive review of late pregnancy tests, including ultrasound parameters to predict adverse pregnancy outcomes, including pre-eclampsia and fetal growth restriction. This work will build on a Cochrane review assessing the diagnostic accuracy of biochemical tests of placental function versus ultrasound assessment of fetal size for stillbirth and SGA infants and will allow us to extend their findings to include data relating to pre-eclampsia and FGR prediction and assess additional data on delivery of the SGA infant and stillbirth, published subsequent to the timeframe of that review.

These conditions remain a significant global cause of maternal and neonatal morbidity and mortality. Identifying an accurate predictive tool using either a single or combination of tests in late pregnancy would allow targeted management and possible intervention for high-risk pregnancies, ultimately avoiding adverse outcomes associated with placental disease.

## Supplementary information

**Additional file 1.** PRISMA-P 2015 Checklist.

**Additional file 2.** Draft search strategy to be used for the Medline online database.

**Additional file 3.**

## Data Availability

The datasets generated and/or analyzed during the current study are available from the corresponding author on reasonable request.

## Abbreviations

AUC: Area under (ROC) curve
EFW: Estimated fetal weight
FGR: Fetal growth restriction
FN: False-negative
FP: False-positive
HSROC: Hierarchical summary ROC
NGR: Neonatal growth restriction
ROC curve: Receiver operating characteristic curve
SGA: Small for gestational age
TN: True-negative
TP: True-positive
UAPI: Umbilical artery Doppler pulsatility index
UtAPI: Uterine artery Doppler pulsatility index
