# Supplementary material for: The ability of late pregnancy maternal tests to predict adverse pregnancy outcomes associated with placental dysfunction (specifically fetal growth restriction and pre-eclampsia): a protocol for a systematic review and meta-analysis of prognostic accuracy studies
Source: Syst Rev. 2020 Apr 8;9:78. doi: 10.1186/s13643-020-01334-5 (PMC7140577; doi:10.1186/s13643-020-01334-5)
Supplement: Supplementary file 2 — Additional file 2. Draft search strategy to be used for the Medline online database. [file 13643_2020_1334_MOESM2_ESM.docx]

Additional File 2

Draft search strategy to be used for the EMBASE online database

| **Search query:** Assessing the prognostic accuracy studies to evaluate the ability of late pregnancy maternal tests to predict adverse pregnancy outcomes associated with placental dysfunction | |
| --- | --- |
| **Sources searched: EMBASE** | |
| **Limits: None**   - Date: - Language: | |
| 1 | (pre eclampsia OR preeclampsia).ti,ab |
| 2 | exp "BIOLOGICAL MARKER"/ |
| 3 | exp STILLBIRTH/ |
| 4 | (stillbirth OR stillborn).ti,ab |
| 5 | (biomarker OR biological marker).ti,ab |
| 6 | exp "PLACENTAL GROWTH FACTOR"/ |
| 7 | (birth weight OR fetal growth OR small for gestational).ti,ab |
| 8 | exp "BIRTH WEIGHT"/ |
| 9 | exp "FETUS GROWTH"/ OR exp "FETUS GROWTH RETARDATION"/ |
| 10 | exp "SMALL FOR DATE INFANT"/ OR exp "SMALL FOR GESTATIONAL AGE"/ |
| 11 | (growth restriction).ti,ab |
| 12 | (oestradiol).ti,ab |
| 13 | (estradiol).ti,ab |
| 14 | exp ESTRADIOL/ |
| 15 | (oestriol).ti,ab |
| 16 | exp PROGESTERONE/ |
| 17 | (progesterone).ti,ab |
| 18 | exp PREGNENOLONE/ |
| 19 | (pregnenolone).ti,ab |
| 20 | exp CHORIONIC GONADOTROPIN/ |
| 21 | (human chorionic gonadotrophin).ti,ab |
| 22 | (hCG).ti,ab |
| 23 | exp PLACENTAL LACTOGEN/ |
| 24 | (hPL).ti,ab |
| 25 | (human placental lactogen).ti,ab |
| 26 | (human placental growth hormone).ti,ab |
| 27 | (placental protein 13).ti,ab |
| 28 | (placental growth factor).ti,ab |
| 29 | (Soluble FMS like tyrosine kinase).ti,ab |
| 30 | (sFlt, OR s-Flt OR s-flt).ti,ab |
| 31 | (Endoglin).ti,ab |
| 32 | (S-Eng).ti,ab |
| 33 | (Vascular endothelial growth factor).ti,ab |
| 34 | (VEGF).ti,ab |
| 35 | (pregnancy specific glycoprotein*).ti,ab |
| 36 | exp PREGNANCY-SPECIFIC BETA 1-GLYCOPROTEINS/ |
| 37 | (schwangerschaft protein 1).ti,ab |
| 38 | (pregnancy specific beta 1-glycoprotein).ti,ab |
| 39 | exp ULTRASONOGRAPHY, PRENATAL/ |
| 40 | (sonograph* OR ultraso*).ti,ab |
| 41 | (1 or 3 or 4 or 7 or 8 or 9 or 10 or 11) |
| 42 | (2 or 5 or 12 or 13 or 14 or 15 or 16 or 17 or 18 or 19 or 20 or 21 or 22 or 23 or 24 or 25 or 26 or 27 or 28 or 29 or 30 or 31 or 32 or 33 or 34 or 35 or 36 or 37 or 38 or 39 or 40) |
| 43 | (41 AND 42) |
