# Supplementary material for: The ability of late pregnancy maternal tests to predict adverse pregnancy outcomes associated with placental dysfunction (specifically fetal growth restriction and pre-eclampsia): a protocol for a systematic review and meta-analysis of prognostic accuracy studies
Source: Syst Rev. 2020 Apr 8;9:78. doi: 10.1186/s13643-020-01334-5 (PMC7140577; doi:10.1186/s13643-020-01334-5)
Supplement: Supplementary file 3 — Additional file 3. [file 13643_2020_1334_MOESM3_ESM.docx]

**Modified QUADAS-2**

**Paper title:**

**Phase 1: State the review question:**


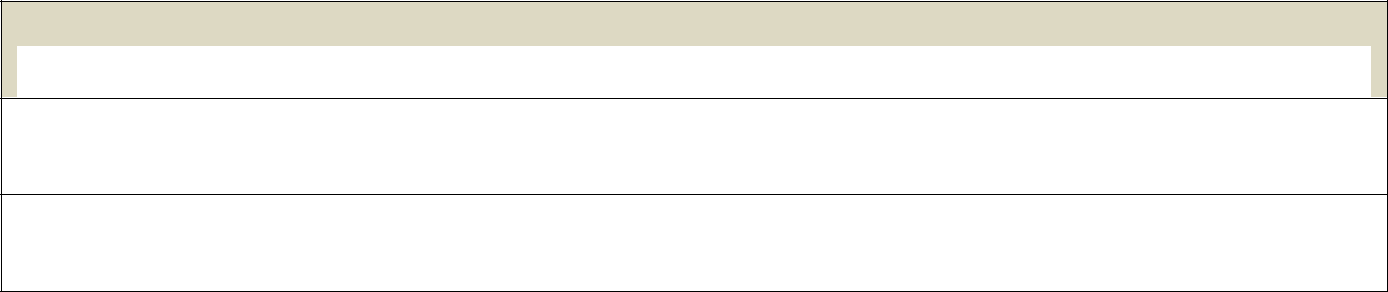


*Patients (setting, intended use of index test, presentation, prior testing):*


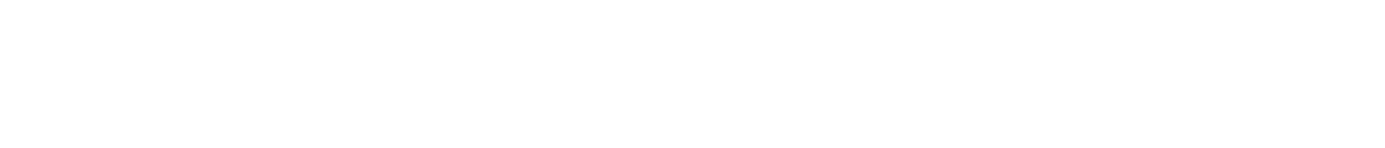

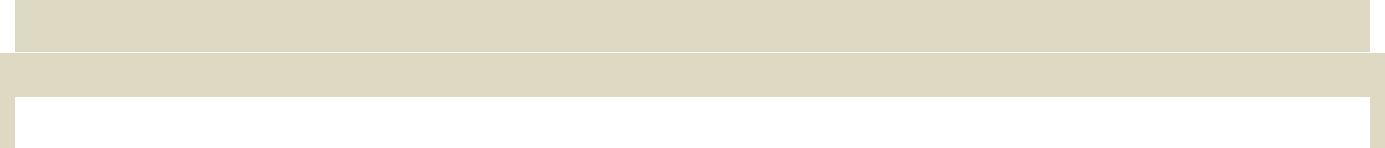


*Index test(s):*


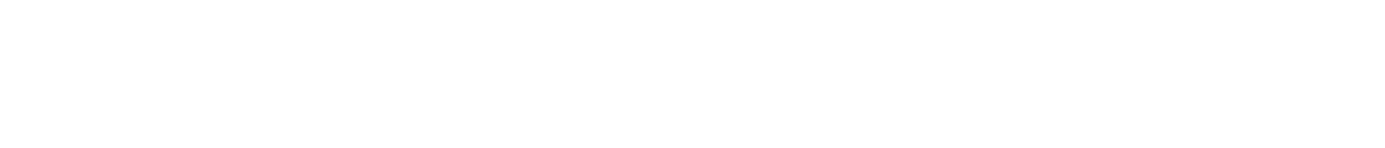

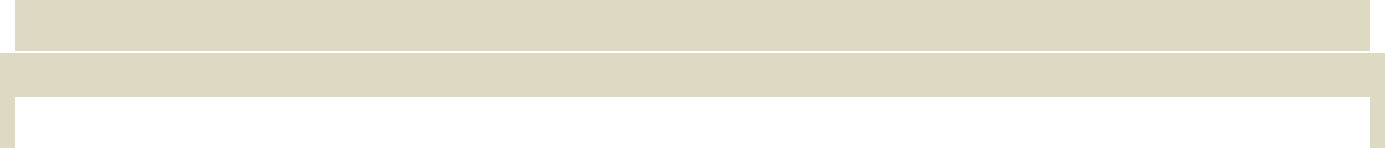


*Reference standard and target condition:*


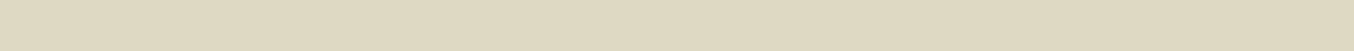


**Phase 2: Draw a flow diagram for the primary study**

**Phase 3: Risk of bias and applicability judgments**

**DOMAIN 1: PATIENT SELECTION**

**A. Risk of Bias**

Describe methods of patient selection:

Was a consecutive or random sample of patients enrolled? Yes/No/Unclear

Was a case-control design avoided (excluding nested case-control)? Yes/No/Unclear

Did the study avoid inappropriate exclusions? Yes/No/Unclear

**Could the selection of patients have introduced bias?** **RISK: LOW/HIGH/UNCLEAR**

**B. Concerns regarding applicability**

Describe included patients (prior testing, presentation, intended use of index test and setting)**:**

**Is there concern that the included patients do not match CONCERN: LOW/HIGH/UNCLEAR the review question?**

**DOMAIN 2: INDEX TEST(S)**

**If more than one index test was used, please complete for each test.**

**A. Risk of Bias**

Describe the index test and how it was conducted and interpreted:

Were the index test results interpreted without Yes/No/Unclear

knowledge of the results of the reference standard?

If a threshold was used, was it pre-specified? Yes/No/Unclear

**Could the conduct or interpretation of the index test** **RISK: LOW /HIGH/UNCLEAR**

**have introduced bias?**

**B. Concerns regarding applicability**

**Is there concern that the index test, its conduct, or** **CONCERN: LOW /HIGH/UNCLEAR**

**interpretation differ from the review question?**

**DOMAIN 3: REFERENCE STANDARD**

**A. Risk of Bias**

Describe the reference standard and how it was conducted and interpreted:

Is the reference standard likely to correctly classify the target Yes/No/Unclear

condition?

Were the reference standard results interpreted without Yes/No/Unclear

knowledge of the results of the index test?

**Could the reference standard, its conduct, or its** **RISK: LOW /HIGH/UNCLEAR**

**interpretation have introduced bias?**

**B. Concerns regarding applicability**

**Is there concern that the target condition as defined by the reference standard does not match the review question?**

**CONCERN: LOW /HIGH/UNCLEAR**

**DOMAIN 4: FLOW AND TIMING**

**A. Risk of Bias**

Describe any patients who did not receive the index test(s) and/or reference standard or who were excluded from the 2x2 table (refer to flow diagram):

Describe the time interval and any interventions between index test(s) and reference standard:

Was there an appropriate interval between index test(s) Yes/No/Unclear

and reference standard?

Did all patients receive a reference standard? Yes/No/Unclear

Did patients receive the same reference standard? Yes/No/Unclear

Were all patients included in the analysis? Yes/No/Unclear

**Could the patient flow have introduced bias?** **RISK: LOW /HIGH/UNCLEAR**
